# Necrobiosis Lipoidica Diabeticorum

**DOI:** 10.1155/2012/152602

**Published:** 2012-04-22

**Authors:** Andrea Scaramuzza, Maddalena Macedoni, Gian Luca Tadini, Laura De Angelis, Francesca Redaelli, Alessandra Gazzarri, Valentina Comaschi, Elisa Giani, Gian Vincenzo Zuccotti

**Affiliations:** ^1^Department of Pediatrics, University of Milano, “Ospedale Luigi Sacco”, Via G.B. Grassi 74, 20154 Milan, Italy; ^2^Department of Dermatology, University of Milano, Via Pace, 4, 20150 Milan, Italy

## Abstract

Necrobiosis lipoidica is a rare disorder that usually appears in the lower extremities and it is often related to diabetes mellitus. There are few reported cases of necrobiosis lipoidica in children. We present an interesting case in that the patient developed lesions on the abdomen, which is an unusual location.

## 1. Case Report

**Figure 1:**
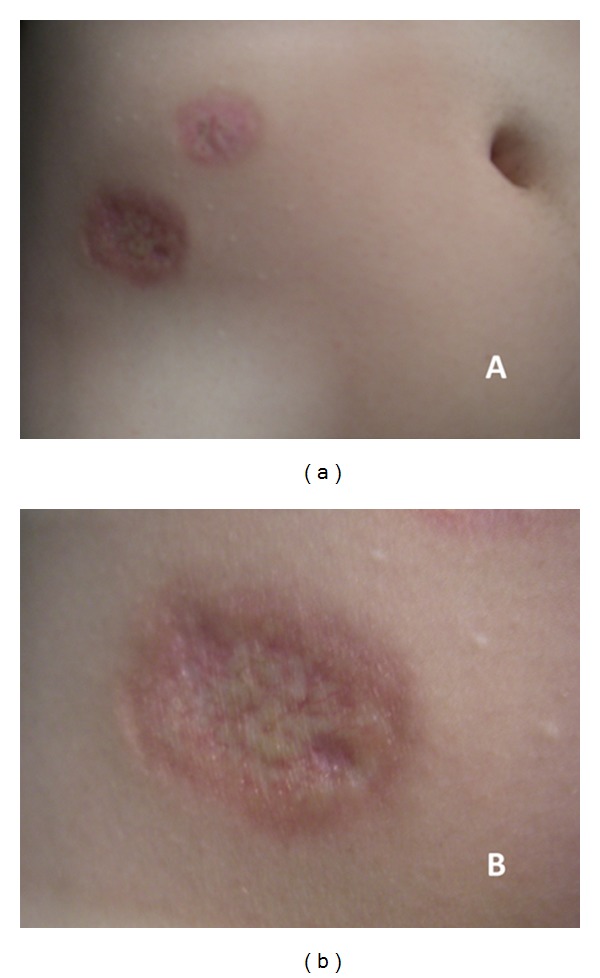
Yellow-brown, atrophic plaques surrounded by raised, violaceus rims on the right side of the lower abdomen (a); particular of the skin lesions (b).

**Figure 2:**
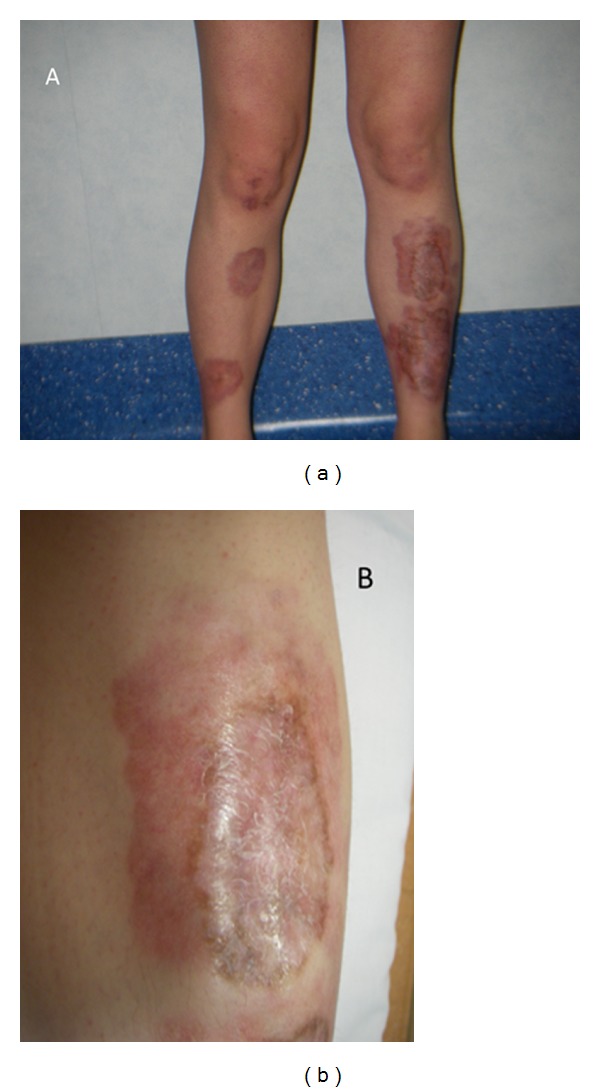
Skin lesions typically appear on the legs (a); particular of the skin lesions (b).

A 16-years-old girl, with type 1 diabetes diagnosed elsewhere in 2000, was brought to our Department for a first routine visit in 2006, during which two yellow-brown, atrophic plaques surrounded by raised, violaceus rims on the right side of the lower abdomen (0.79 × 0.2 in and 1.38 × 0.79 cm, resp.) (Figure 1) were observed. Several other plaques had been observed on both legs (Figure 2). No other significant finding had been seen. A careful history revealed that she was on bad terms with her diabetes. Since diagnosis she had a multiple daily injections (MDIs) scheme for her insulin therapy (mean insulin requirement: 0.97 U/kg/day). Due to poor glycemic control she was switched to insulin pump therapy in 2006. Despite a slight improvement in glycemic values and reduction in insulin requirement (0.70 U/kg/day), her glycated hemoglobin (HbA1c) remained high (mean ± SD: 12.9 ± 1.1), without any improvement of the skin lesions. In 2008 she returned to MDI, and her last HbA1c (December 2010) was 12.8%, with an increasing insulin requirement (1.58 U/kg/day). Topical steroids were unhelpful in improving skin lesions.

## 2. Discussion

Necrobiosis lipoidica (NL) is an idiopathic dermatological condition that is strongly associated with diabetes mellitus, so much so that some authors name it “diabeticorum.”

NL is characterized by a rash that occurs on the lower legs and only rarely on hands, fingers, face, and scalp [1]. It is noteworthy that, in our patient, beyond the usual presentation on the legs, two skin lesions appeared on the lower part of the abdomen.

At the beginning, the lesions appeared as erythematous circle papules that then evolve to well-demarcated, atrophic, shiny, yellow-brown telangiectasic multiple and bilateral plaques. Except as they are ulcerated, the lesions are generally asymptomatic [2]. NL is more common in women than in men [1], and it appears usually in young or middle adulthood [3]; few cases have been, however, reported in childhood [4]. NL prevalence varies from 0.3% to 1.2% among diabetic patients [2], two-thirds of whom have type 1 diabetes.

The pathogenesis of NL is largely enigmatic. It has been suggested that NL is one of the possible manifestations of microangiopathy, due to its clear association with diabetes. Some authors [5] suggest that in patients with diabetes NL might be a warning sign for nephropathy and retinopathy. Whether or not poor glucose control is associated with the development and progression of NL lesion remains controversial [6]. Despite its possible role in NL pathogenesis, tight glycemic control might prevent NL or even improve skin lesions, when present [6].

Few data exist in pediatric population. A recent paper by Pavlović et al. [7] reported an NL incidence of 2.3%, higher than that reported in the literature; there are 5 patients (4 females and 1 male) out of 212 patients with type 1 diabetes (mean age 14 years, mean disease duration 7 years); no significant relationship has been observed with disease duration, age, glycemic control. After a review of our patients, we have found 3 patients with NL out of 240 (1.25%), 2 females and 1 male. All of them have poor glycemic control (mean HbA1c: 13.4 ± 2.1%), initial sign of microangiopathic complications (2 nephropathy, 3 neuropathy, 1 retinopathy). However, glycemic control per se is not suggestive of NL appearance. Indeed, in our population only 3 patients out of the about 30 with a steady HbA1c value above 9% developed NL, confirming that other factors have to be involved in NL pathogenesis. The finding of IgM, IgG, IgA, and C3 in blood vessel walls might support the role of immunological-mediated vascular disease in NL.

Some authors [8] regard NL to be a primary disease of collagen, with inflammation occurring as a second event. For this reason a strict avoidance of trauma is suggested to prevent ulceration and the development of new lesions.

NL diagnosis is commonly a clinical one; only in those few cases, usually in the starting stages of the disease, when the diagnosis may be awkward, a biopsy of the lesion can be useful.

Treatment of NL is often difficult. First-line therapies include smoking cessation and diabetes control improvement. In addition, topical and intralesional corticosteroid may be effective [9], even if they can increase glycemic values. In the present case, no improvement of the skin lesions has been observed despite the use of topical corticosteroid. A possible explanation could be the steady bad glycemic control of the patient.

Recently, a new therapeutic attempt has been reported, including topical PUVA photochemotherapy [10]: after a mean of 47 sessions all 10 treated patients experienced almost complete remission of the skin lesions.

On the other side, systemic therapies using corticosteroids and azathioprine could facilitate malignant transformation [11]. Squamous cell carcinomas have been reported to arise in areas of NL [11].

Differential diagnosis includes granuloma annulare, sarcoidosis, and amyloidosis. Granuloma annulare has typical lesions with epidermis not thinned. Besides the histopathologic findings that may rule out the differences between the diseases, it shows resolution with near restoration of structure, normal and without sclerosis. On the other hand, necrobiosis lipoidica shows a progression to collagen bundles in the reticular dermis in septa in the subcutaneous fat becoming crowded and thickened. Sarcoidosis is a multisystem disease that may involve almost any organ system, and its dermatological findings can assume a vast array of morphologies. Correctly diagnosing sarcoidosis may be a challenge. Patients are diagnosed with sarcoidosis when a compatible clinical or radiologic picture is present, along with histologic evidence of noncaseating granulomas, and when other potential causes, such as infections, are excluded. Amyloidosis is a systemic disease too, a plasma-cell dyscrasia of unknown cause, with well-recognized dermatological signs that may be the presenting features. Skin and soft-tissue lesions may, indeed, be the only manifestations of the disease prior to later-stage organ involvement. Although the skin manifestations of systemic amyloid are common to many conditions, their presentation in certain clinical settings should help to indicate this disorder for inclusion in the differential diagnosis.
